# Spontaneous Perforation of Meckel’s Diverticulum in a 37-Year-Old Woman: A Case Report

**DOI:** 10.7759/cureus.95304

**Published:** 2025-10-24

**Authors:** Ryan Darayseh, Abdulrahman J Aakef, Aysha Al Shamsi, Khadeeja A, Hamda Al Shehhi, Abdullah O Al Ani, Saabh I Khalil

**Affiliations:** 1 Internal Medicine, Ajman University, Ajman, ARE; 2 Neurosurgery, Ajman University, Ajman, ARE; 3 Pediatrics, Ajman University, Ajman, ARE; 4 Medicine, Mohammed Bin Rashid University of Medicine and Health Sciences, Dubai, ARE; 5 Surgery, Ajman University, Ajman, ARE; 6 Radiology, Ajman University, Ajman, ARE; 7 Radiology, Sheikh Khalifa Hospital, Ajman, ARE

**Keywords:** acute abdomen, diagnostic laparoscopy, exploratory laparotomy, ileal perforation, meckel’s diverticulum, peritonitis, segmental small bowel resection, small bowel perforation

## Abstract

Introduction: Meckel’s diverticulum (MD), the most common congenital abnormality of the gastrointestinal (GI) tract, typically presents in an asymptomatic manner. However, peritonitis occurring as a result of ruptured MD is extremely rare in adults, and this may make diagnosis even more challenging. This presentation can often imitate other acute GI emergencies like appendicitis or even some gynecological conditions.

Case presentation: We report an unusual case of a 37-year-old female patient presenting with lower abdominal pain radiating to the right iliac fossa for three days, progressing to generalized abdominal pain along with signs of peritonitis. On exploratory laparotomy, findings included a severely congested and edematous MD with a large 1 cm perforation, as well as a mass of hard tissue within its wall. Segmental enterectomy with primary end-to-end anastomosis and an appendectomy was performed.

Discussion: The differential diagnosis of ruptured MD should never be overlooked in adult patients presenting with acute abdominal pain and generalized peritonitis, even though it is very rare. Diagnosis of this disease preoperatively is extremely complex due to the fact that radiological findings are usually not specific and may imitate other acute abdominal or gynecological emergencies. For this reason, immediate surgical intervention is crucial to prevent complications such as widespread sepsis and chronic illness.

Conclusion: This case emphasizes the importance of keeping the differential of ruptured MD under consideration when dealing with adults presenting with acute abdominal pain, even if a gynecological cause is initially suspected. Thus, early detection and immediate surgical intervention are key factors for more positive patient outcomes.

## Introduction

Acute abdomen is a common clinical presentation housing a wide range of possible underlying causes, some of which are self-limiting and others that are life-threatening and require immediate intervention. In women of reproductive age, obstetric and gynecological etiologies should be considered, including ruptured ovarian cysts or ectopic pregnancies, which have been increasingly seen. However, surgical differentials must not be overlooked, especially when patients present with signs of peritonitis, suggesting the urgency of the care needed. In addition, congenital anomalies should also be considered as they can present acutely and severely [1].

In this respect, Meckel's diverticulum (MD) is the most common congenital malformation of the gastrointestinal tract, occurring in about 2-3% of the general population. MD may occur in a small number of people; however, only 4-6% will develop symptoms during their lifetime, and the majority of patients will have their first episode of symptomatology during childhood. The most common complications resulting from Meckel's diverticulum include intestinal obstruction (40%), gastrointestinal bleeding (30%), and inflammation or perforation (20%). In adults, MD perforation is rare and has, until now, been unheard of. Therefore, this case report describes a rare instance of perforated and ruptured MD in an adult woman. This current endeavor will seek to highlight the need for a wide differential diagnosis amongst adult patients with acute abdomen and an investigation into the problems with diagnosing patients with MD [1].

## Case presentation

A 37-year-old female patient presented to the emergency department at around 10 pm with complaints of lower abdominal pain since yesterday and constipation for one week. The patient did not exhibit any other symptoms and refused further investigation or medication, opting instead to sign the leave-against-medical-advice (LAMA) and leave.

The patient presented the next day to the emergency department at 7 pm with the same complaints. The abdominal pain had progressed into severe pain along with one episode of non-bilious, non-bloody vomiting, with on/off nausea and dizziness upon standing. The pain initially started around the right iliac fossa, and now it has become generalized to the entire abdomen. The patient stated that the pain began suddenly and described the character of the pain as a sharp, constant pain without any relieving or aggravating factors. History revealed no previous similar episodes. She stated that she is not married and is not sexually active. Surgical history revealed a laparoscopic sleeve gastrectomy performed three years prior for weight reduction. As the surgery primarily involves the upper abdomen, any postoperative adhesions would likely be limited to that region and therefore unlikely to contribute to the current lower abdominal presentation or delay diagnosis. Family history, social history, and gynecological history were unremarkable.

On physical examination, she was afebrile (36.9 °C), borderline hypotensive (BP 97/59 mmHg); all other vitals are normal (heart rate (HR) 70 bpm, respiratory rate (RR) 18 bpm, SpO_2_ 100% on room air, height 156 cm, weight 55 kg, body mass index (BMI) 22.6 kg/m^2^). On general examination, the patient was conscious, oriented (Glasgow Coma Scale (GCS) 15/15), and slightly pale. On the abdominal examination, the abdomen was non-distended, with multiple healed laparoscopic scars, without signs of visible hernias or masses. Upon palpation, the patient experienced generalized tenderness, guarding, and rebound tenderness along with tenderness and dullness on percussion with hyperactive bowel sounds on auscultation.

Laboratory investigations revealed mild leukocytosis (12.17×10^9^/L) with neutrophilia (absolute neutrophil count (ANC) 11.94×10^9^/L), significantly elevated C-reactive protein (CRP) (204 mg/l), mildly elevated procalcitonin (0.23 ng/ml), with prolonged prothrombin time (PT) (19.2s) and activated partial thromboplastin time/partial thromboplastin time (APTT/PTT) (43.5s), mildly elevated international normalized ratio (INR) (1.4) and slightly low hemoglobin (HB) count (11.6 g/dl).

**Table 1:** Patient's vitals and laboratory investigations on admission. This table summarizes the patient’s vital signs and laboratory investigations on admission, along with the corresponding reference ranges. SpO_2_: peripheral capillary oxygen saturation; CRP: C-reactive protein; PT: prothrombin time; APTT/PTT: activated partial thromboplastin time/partial thromboplastin time; INR: international normalized ratio, Hb: hemoglobin.

Labs	Results	Reference range
Temperature	36.9 °C	36.1 – 37.9 °C
Blood pressure	97/59 mmHg	90/60 – 120/80 mmHg
Heart rate	70 bpm	60 – 100 bpm
Respiratory rate	18 bpm	12 – 20 bpm
SpO_2_	100%	95 – 100%
Leukocyte	12.17×10⁹/L	(4.0 – 10) x10⁹/L
Neutrophils	11.94×10⁹/L	(2.0 – 7.5) x10⁹/L
CRP	204 mg/l	<5 mg/L
Procalcitonin	0.23 ng/ml	<0.05 ng/ml
PT	19.2	11 – 13.5 s
APTT/PTT	43.5	25 – 35 s
INR	1.4	0.8 – 1.2
Hb	11.6 g/dl	12 – 16 g/dL

The initial radiological investigations included a transabdominal sonography (TAS), which showed a normal uterus with thick endometrial thickness (ET), a normal left ovary, and a collapsed cyst in the right ovary, with a moderate amount of free fluid surrounding the uterus and in both paracolic gutters. A computerized tomography (CT) scan with intravenous (IV) contrast of the abdomen revealed mild to moderate intraperitoneal free fluid on the right side of the abdomen, in the intrapelvic cul-de-sac, and right adnexa, along with a collapsed right ovarian cyst and no signs of bowel perforation. These findings initially suggested a ruptured ovarian cyst.

**Figure 1:**
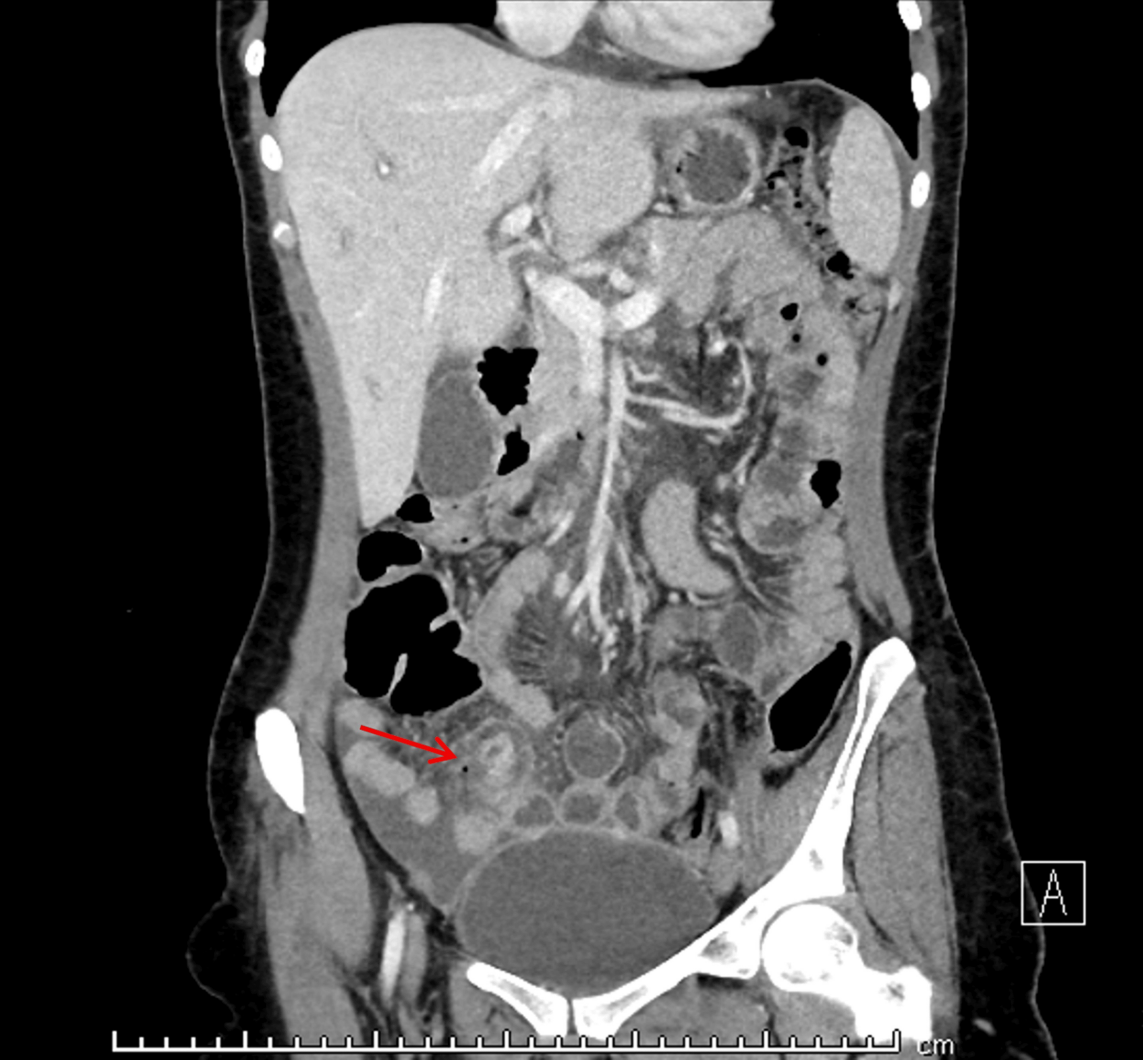
Coronal section contrast-enhanced CT showing perforated Meckel’s diverticulum (interrupted wall). The red arrow is pointing to a blind-ending, tubular structure arising from the distal ileum, with focal wall thickening and discontinuity, surrounded by fat stranding and inflammatory changes. This radiologic appearance is consistent with a perforated Meckel’s diverticulum.

**Figure 2:**
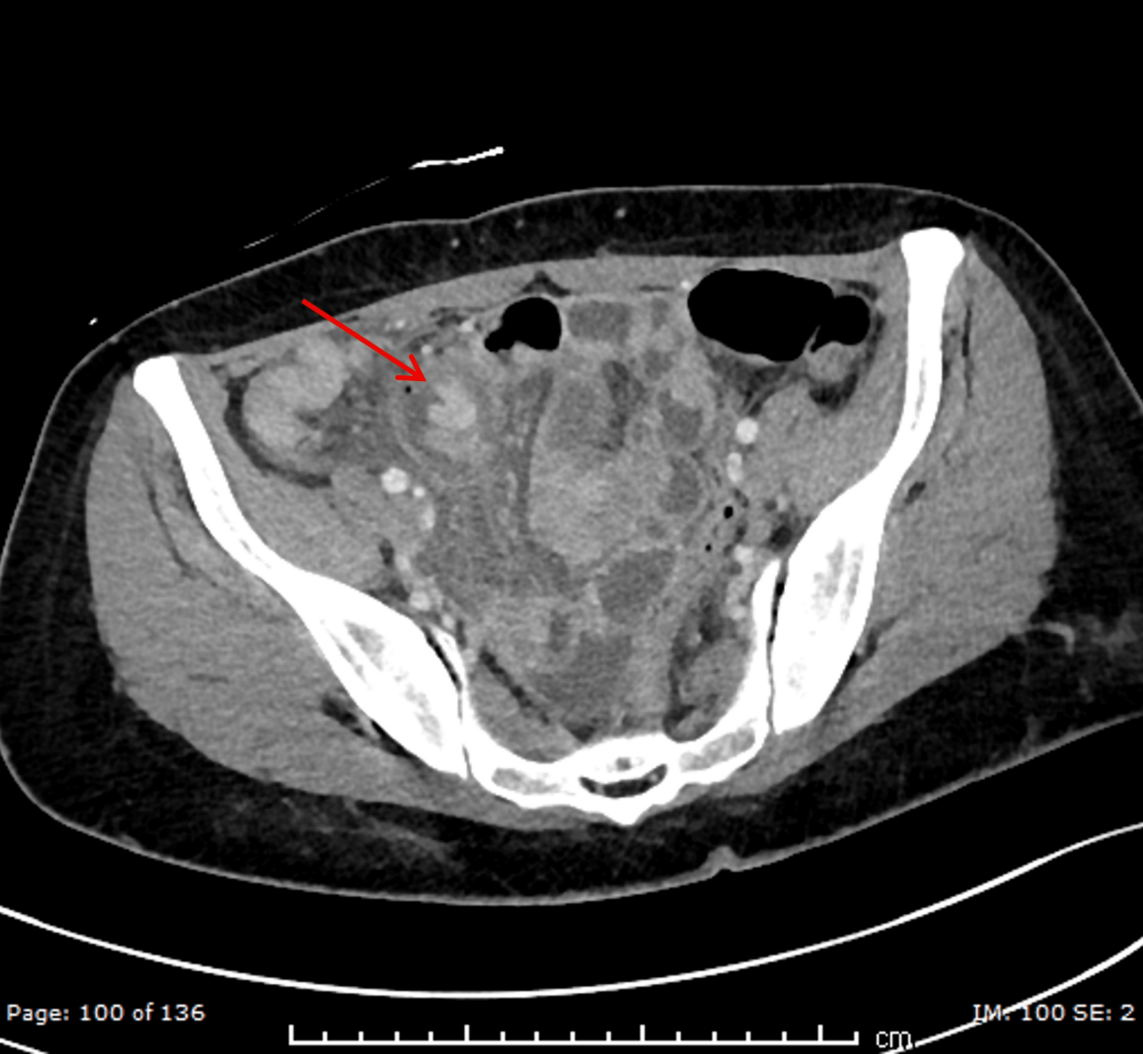
Axial section contrast-enhanced CT showing Meckel’s diverticulum, along with localized air nearby suggesting perforation. The red arrow is pointing to a thick-walled, blind-ending tubular structure arising from the distal ileum with focal wall interruption and adjacent fat stranding, consistent with perforated Meckel’s diverticulum.

Upon further review of the CT images, however, additional abnormalities were identified. The coronal section demonstrated a blind-ending tubular structure arising from the distal ileum with focal wall thickening and discontinuity, surrounded by fat stranding and inflammatory changes, consistent with a perforated MD (Figure 1). The axial section further highlighted the diverticulum with adjacent localized air, again suggesting perforation (Figure 2).

The patient was admitted to the maternity ward and initially treated conservatively (nil per os (NPO), IV fluids, and regular monitoring of vital signs). She was subsequently transferred to the surgical ward and prepared for diagnostic laparoscopy, with a high likelihood of conversion to laparotomy. Preoperative management included antibiotic prophylaxis with ciprofloxacin 100 mL (200 mg IV q12hr, infused over 60 minutes) and a repeat electrolyte panel.

The diagnostic laparoscopy revealed no blood in the abdomen, but pus and fluid were all over the peritoneal cavity in addition to multiple adhesions. The uterus and ovaries were examined, and no abnormalities were found. The small intestine examination showed omentum adherent to the site of the perforated MD. A decision was then made to convert to an exploratory laparotomy with peritoneal lavage, revealing a diverticulum 60 cm from the ileocecal junction, around 15 cm of distal ileum containing the diverticulum, which was severely congested and edematous (3 cm diameter), including a big 1 cm perforation along with a hard tissue mass in its wall. The small intestinal loop was then isolated and resected with an end-to-end anastomosis of the distal ileum.

The appendix was congested, and pus was found in the abdominal cavity with adhesions surrounding the perforated MD, leading to the decision to perform an appendectomy. The histopathological examination confirmed acute appendicitis, unlike the Meckel’s specimen, which could not be retrieved for histopathological review.

The patient had an uneventful recovery after surgery and was in stable condition when discharged on postoperative day five. She was advised to return in two weeks for postoperative follow-up, but did not attend, so no information about long-term data was available.

## Discussion

While MD is the most common congenital anomaly of the gastrointestinal tract, affecting roughly 2-3% of the population [2-3], its complications are rarely seen in adults [3], particularly in women. Sagar et al. report that only a small percentage of symptomatic Meckel’s cases involve perforation, and most occur in males [3]. This case adds to the limited number of reported adult female presentations, and even more rarely, one complicated by perforation and generalized peritonitis [4].

The diagnostic dilemma in this case arose from the nonspecific clinical and radiological findings [5]. This nonspecific presentation resembled more common conditions such as appendicitis [6-11] or a ruptured ovarian cyst, especially since initial imaging only revealed free fluid and a suspected bowel wall irregularity [10]. These findings are not uncommon in cases of Meckel’s perforation, where even contrast-enhanced CT only demonstrates nonspecific signs [6] and lacks specificity. Similar reports have emphasized that in adults, symptomatic MD is frequently misdiagnosed or only recognized intraoperatively because of overlapping imaging features and its rarity [9]. Oricchio et al. emphasize that diagnosis is rarely made preoperatively and often mimics other causes of acute abdomen [8]. As seen in many similar cases, a definitive diagnosis was only made intraoperatively [7-8], highlighting the difficulty of diagnosing MD based solely on preoperative imaging. Another review by Fraser et al. also concluded that preoperative diagnosis is extremely uncommon, and almost all adult cases of perforated Meckel’s are diagnosed intraoperatively [4]. This further supports the importance of early surgical exploration in cases of unexplained acute abdominal pain.

A critical aspect of this case was the identification of dense intra-abdominal adhesions. These adhesions could have been misattributed to the patient’s prior sleeve gastrectomy. However, intraoperative findings localized them to the site of the perforated Meckel’s diverticulum. With this localization and the documented presence of pus found throughout the peritoneal cavity, it was confirmed that the adhesions were reactive, developing in response to local inflammation.

Our case also highlights the clinical ambiguity frequently encountered in women of childbearing age presenting with acute abdominal pain [8]. The preliminary assessment strongly suggested a gynecological etiology, prompting consultation with both gynecology and general surgery. However, the eventual diagnosis of a surgical pathology reinforces the importance of considering a broad differential. Surgical etiologies should not be underestimated, even when early clinical signs, laboratory investigations, or diagnostic imaging point toward a gynaecologic condition.

In conclusion, this case highlights the intraoperative difficulties involved with complicated MD. Although laparoscopic treatment is increasingly documented in cases of uncomplicated MD, conversion to open laparotomy remains a safer option in the setting of perforation, extensive adhesions, or peritoneal contamination [10].

## Conclusions

To conclude, this report elucidates the diagnostic complexity and potential clinical severity of clinically manifesting ruptured MD in adult women. In addition, it emphasizes the importance of considering an extensive diagnostic scope in the diagnostic process of unexplained peritonitis, as rare and infrequent conditions may present with deceptive clinical signs or non-classical features. This case emphasizes the challenge of preoperative diagnosis, as imaging findings are often nonspecific, and definitive identification usually occurs intraoperatively. In addition, the case highlights the importance of making an urgent decision to operate. Laparoscopic management is preferred in uncomplicated cases, but conversion to laparotomy is important if perforation is found, dense adhesions are found, and/or the peritoneal cavity is contaminated.

Timely surgical treatment is critical for reducing the chance of deterioration in a clinical state, especially when initial evaluations and preoperative investigations are inconclusive. Understanding these diagnostic and procedural nuances may help clinicians to manage the presentation more effectively and avoid possible morbidity associated with delayed intervention.
